# Hypoxia-Inducible Factors Activate CD133 Promoter through ETS Family Transcription Factors

**DOI:** 10.1371/journal.pone.0066255

**Published:** 2013-06-20

**Authors:** Shunsuke Ohnishi, Osamu Maehara, Koji Nakagawa, Ayano Kameya, Kanako Otaki, Hirotoshi Fujita, Ryosuke Higashi, Kikuko Takagi, Masahiro Asaka, Naoya Sakamoto, Masanobu Kobayashi, Hiroshi Takeda

**Affiliations:** 1 Department of Gastroenterology and Hepatology, Hokkaido University Graduate School of Medicine, Sapporo, Japan; 2 Department of Pathophysiology and Therapeutics, Faculty of Pharmaceutical Sciences, Hokkaido University, Sapporo, Japan; 3 Department of Cancer Preventive Medicine, Hokkaido University Graduate School of Medicine, Sapporo, Japan; 4 Department of Nursing, Health Sciences University of Hokkaido, Ishikari, Japan; University of Nebraska Medical Center, United States of America

## Abstract

CD133 is a cellular surface protein that has been reported to be a cancer stem cell marker, and thus it is considered to be a potential target for cancer treatment. However, the mechanism regulating CD133 expression is not yet understood. In this study, we analyzed the activity of five putative promoters (P1–P5) of CD133 in human embryonic kidney (HEK) 293 cells and colon cancer cell line WiDr, and found that the activity of promoters, particularly of P5, is elevated by overexpression of hypoxia-inducible factors (HIF-1α and HIF-2α). Deletion and mutation analysis identified one of the two E-twenty six (ETS) binding sites (EBSs) in the P5 region as being essential for its promoter activity induced by HIF-1α and HIF-2α. In addition, a chromatin imunoprecipitation assay demonstrated that HIF-1α and HIF-2α bind to the proximal P5 promoter at the EBSs. The immunoprecipitation assay showed that HIF-1α physically interacts with Elk1; however, HIF-2α did not bind to Elk1 or ETS1. Furthermore, knockdown of both HIF-1α and HIF-2α resulted in a reduction of CD133 expression in WiDr. Taken together, our results revealed that HIF-1α and HIF-2α activate CD133 promoter through ETS proteins.

## Introduction

A growing body of evidence supports the idea that a small fraction of undifferentiated cells are involved in initiating and sustaining tumor growth. Those cells are referred to as cancer stem cells (CSCs) [1], and CD133 is considered to be a CSC markers in tumors of various tissues including the brain [2], prostate [3], liver [4], pancreas [5] and colon [6], [7].

**Figure 1:**
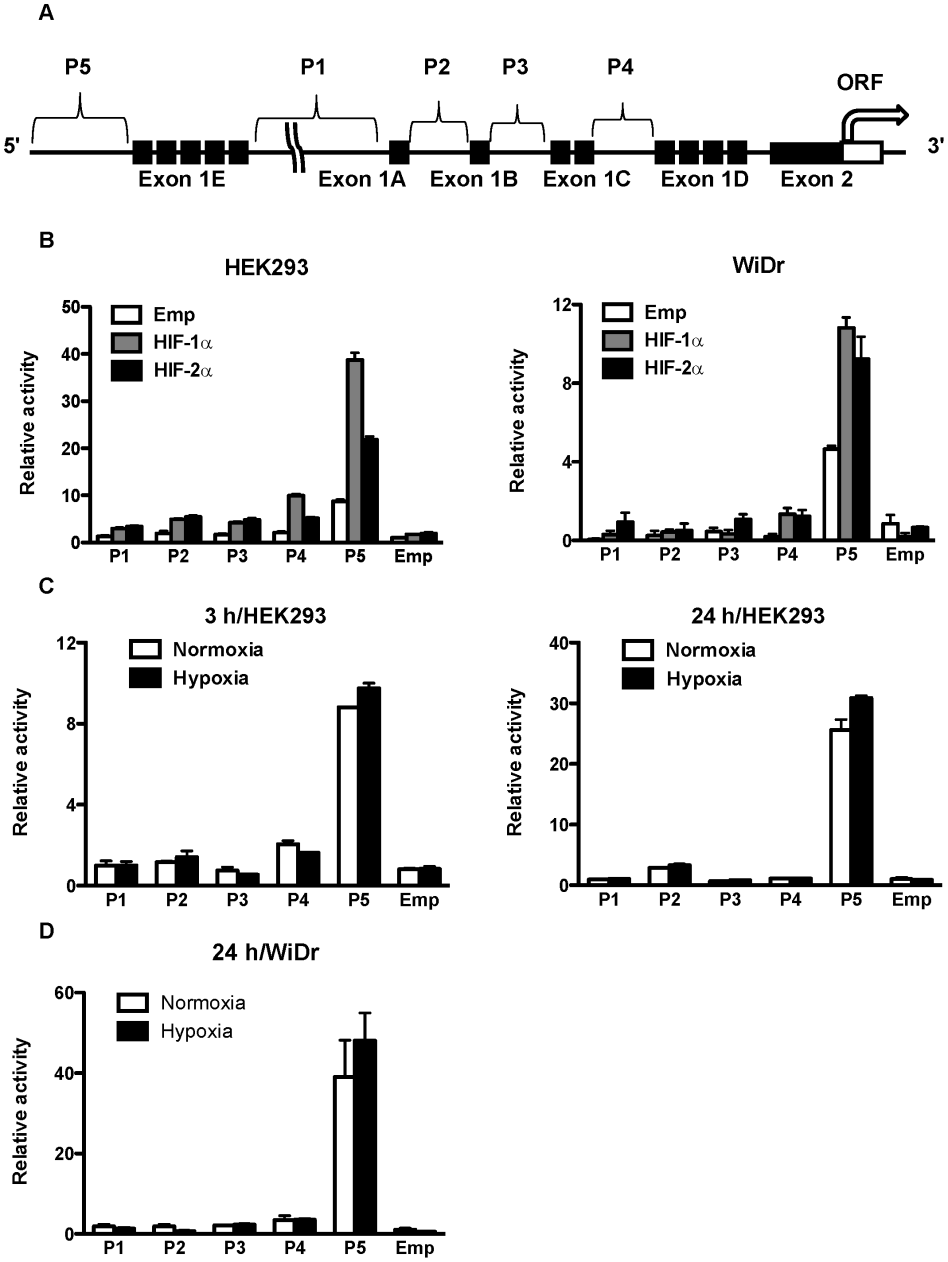
Position of CD133 P5 promoters and their activities after overexpression of HIFs and under hypoxia. (A) Schematic representation of the position of five CD133 promoters (P1–P5) and exon1s (A–E). (B) Promoter activity of P1, P2, P3, P4 and P5 in human embryonic kidney (HEK) 293 cells (left) and human colon cancer WiDr cells (right) after overexpression of HIF-1α and HIF-2α. (C) Promoter activity of P1, P2, P3, P4, and P5 in HEK293 cells under normoxia and hypoxia for 3 hrs (left) and 24 hrs (right). (D) Promoter activity of P1, P2, P3, P4, and P5 in WiDr cells under normoxia and hypoxia for 24 hrs.

CD133 is a cellular surface glycoprotein comprising five transmembrane regions and two glycosylated extracellular loops, and it has a molecular weight of 97–120 kDa [8], [9]. It has been reported that CD133 gene transcription is regulated by five alternative promoters: P1, P2, P3, P4, and P5 (Fig. 1A) [10]. Recent studies have shown that the methylation status of CpG sites in P1 and P2 is involved in epigenetic regulation of CD133 in colorectal and glioblastoma tumors [11], [12]. However, the mechanisms underlying the regulation of CD133 expression are not fully understood.

Increased expression of hypoxia-inducible factors (HIFs) has been documented in many solid tumors, and high expression levels of HIF-1α are usually linked to poor prognosis in cancer patients, including those with colorectal cancer [13]–[15]. HIF is a heterodimer of the basic helix-loop-helix/Per-ARNT-Sim (bHLH-PAS) proteins and comprises of an O_2_-labile α-subunit and β-subunit, acting together to bind to the DNA at specific locations called hypoxia response elements (HREs) [16]. Of the three types of α-subunits, HIF-1α and HIF-2α have been studied most extensively [17].

CSCs have been considered to be dependent on HIF-1α and HIF-2α for survival and tumor growth [18]. In addition, several lines of evidence suggest that HIFs promote the expansion of CD133-positive glioma cells [19] or expression of CD133 in lung cancer cells [20]; however, how the expression of the CSC marker protein is regulated by HIF-1α and HIF-2α remains largely unknown.

Therefore, we investigated the activity of CD133 promoters after overexpression of HIF-1α and HIF-2α, and identified an E-twenty six (ETS) ETS binding site (EBS) as a target of HIF-1α and HIF-2α. Subsequently, we investigated the binding of HIF-1α and HIF-2α at EBS and whether HIF-1α and HIF-2α regulate the expression of CD133 in colon cancer cells.

## Materials and Methods

### Cells

HEK 293 cells (provided by the RIKEN BRC through the National Bio-Resource Project of the MEXT, Japan) and the human colon cancer cell line WiDr (provided by Health Science Research Resources Bank, Osaka, Japan) were cultured in Dulbecco’s modified Eagle’s medium (Wako, Osaka, Japan) supplemented with 10% fetal bovine serum (Invitrogen, Carlsbad, CA), 100 U/ml penicillin and 100 µg/ml streptomycin (Invitrogen), and incubated at 37°C under normoxia (20% O_2_, 5% CO_2_) or hypoxia (1% O_2_, 5% CO_2_).

### Plasmids and Small Interfering RNAs (siRNAs)

Complementary cDNAs of HIF-1α, HIF-2α, ETS1 and Elk1 were obtained by RT-PCR and the resulting fragments were cloned into pCMV-3×FLAG and pCI-neo-2×S vectors, which had been constructed by inserting oligonucleotides encoding 3×FLAG and 2×S tag, into pcDNA3.1 vectors (Invitrogen) and pCI-neo (Promega, Madison, WI, USA), respectively. O_2_-stable mutants of HIF-1α (P402/564A) and HIF-2α (P405/531A) and dominant negative mutants of ETS1 and Elk1 (ETS1-DN; amino acids 350–485, and Elk1-DN; amino acids 1–168, respectively) were generated using a PCR-based method. The CD133 clones comprising promoter fragments P1–P5-Luc and −1368, −768, −368, −25-Luc of P5 were provided by Dr. S. Tanaka of Hokkaido University, Japan [21]. Mutants of two EBSs (EBS1 and EBS2) in the P5 promoter were constructed, by introducing substitutional mutations into ETS core sequence (GGAA to TTAA), using a PCR-based method. siRNAs for HIF-1α and HIF-2α were purchased from Qiagen (Hilden, Germany).

### Transient Transfection and Reporter Gene Assay

Cells were plated at a density of 1×10^5^ cells in 24-well plates containing 500 µl of culture medium. After incubation for 24 hrs at 37°C, cells were transfected with 100 ng luciferase plasmid DNA with 10 ng Renilla pGL4.75 (hRluc-CMV) vector (Promega) as an internal control, using lipofectamine2000 (Invitrogen). A reporter gene assay was performed using the Dual Luciferase reporter assay system (Promega), and the luminescence intensity was measured using an AB-2000 Luminescencer-PSN (Atto, Tokyo, Japan) according to the manufacturer’s protocol. The transcription activity was normalized according to Renilla luciferase activity. Experiments were performed in triplicates.

### Quantitative Real-time Reverse-transcription Polymerase Chain Reaction (qRT-PCR)

Total RNA was extracted from cells using an RNeasy Mini Kit (Qiagen) according to the manufacturer’s instructions. RNA was quantified by spectrometry, and the quality was confirmed by gel electrophoresis. One microgram of total RNA was reverse-transcribed into cDNA using an M-MLV reverse transcriptase (Invitrogen). PCR amplification was performed in 50 µl containing 1 µl cDNA and 25 µl Platinum SYBR Green PCR Mix (Invitrogen). β-actin mRNA amplified from the same samples served as an internal control. After an initial denaturation at 95°C for 2 min, a two-step cycle procedure was used (denaturation at 95°C for 15 sec, annealing and extension at 60°C for 1 min) for 40 cycles in a 7700 Sequence Detector (Applied Biosystems). Gene expression levels were determined using the comparative threshold cycle (ddCt) method with β-actin as an endogenous control. The data were analyzed with Sequence Detection Systems software (Applied Biosystems).

### Western Blot Analysis

Cells were washed with ice-cold Tris-buffered saline (TBS) (-) and lysed in 1×SDS sample buffer containing 2% 2-mercaptoethanol. The samples were heated at 95°C for 5 min and then subjected to sodium dodecyl sulfate-polyacrylamide gel electrophoresis (SDS-PAGE). The separated proteins were transferred to Immobilon-P polyvinylidene difluoride (PVDF) membranes (Millipore, Bedford, MA), which were subsequently incubated in TBS with 0.05% Tween 20 (TBST) comprising 5% dried nonfat milk for 30 min at room temperature. Membranes were probed with primary antibodies for HIF-1α (1∶250, BD Biosciences, Bedford, MA), HIF-2α (1∶200, Santa Cruz Biotechnology, Santa Cruz, CA), CD133 (1∶1,000, Cell Signaling, Danvers, MA), and bound antibodies were detected with peroxidase-labeled rabbit or mouse antibodies (Jackson ImmunoResearch Laboratories, West Grove, PA) and visualized using Immobilon Western horse radish peroxidase (HRP) Substrate detection reagents (Millipore).

### Chromatin Immunoprecipitation (ChIP) Assay

WiDr cells (1.8×10^6^ cells) were plated in 10 cm dish with 10 ml culture medium. After incubation for 24 hrs at 37°C, cells were transfected with 4,000 ng of plasmid encoding FLAG-tagged HIF-1α and HIF-2α. After the incubation period was extended for 48 hrs at 37°C, cells were fixed with 1% formaldehyde and then chromatin was subsequently prepared using the ChIP-IT^®^ Express Kit (Active Motif, Carlsbad, CA) and sheared by sonication. Immunoprecipitation was performed using 15 µg sheared chromatin and 2 µg anti-FLAG antibody (Sigma, St. Louis, MO). DNA collected by ChIP was amplified by qRT-PCR. The target primer was designed for the location of P5 between −98 and +10 (5′-CAGTGTCTCCCCAGAGAG-3′ and 5′-GCAACTTCTACCAGCCTAAGG-3′). For normalization, a control primer was designed at for Exon 2 of CD133 (5′-GGAACACGCTTGCCTTCCCCA-3′ and 5′-CCCAGCAGCAACAGGGAGCC-3′).

### Immunoprecipitation

HEK293 or WiDr cells (5×10^5^ cells, respectively) were plated in 6 cm dishes with 6 ml culture medium. After incubation for 24 hrs at 37°C, cells were transfected with 8,000 ng of plasmid DNA (5,000 ng S-tagged HIF-1α or HIF-2α, and 3,000 ng FLAG-tagged ETS transcription factors). After incubation for 48 hrs at 37°C, cells were lysed in 0.4 ml lysis buffer comprising 20 mM Tris–HCl (pH 7.5), 137 mM NaCl, 0.5% NP-40, 0.5 mM dithiothreitol (DTT), complete protease inhibitor cocktail (Roche, Mannheim, Germany), and phosphatase inhibitor cocktail (Sigma), then pre-cleared by centrifuging at 15,000 rpm for 15 min at 4°C. Supernatant was collected as a whole cell lysate. Immunoprecipitation was performed using FLAG M2 agarose (Sigma), and whole cell lysate (360 µl) was added in 10 µl FLAG M2 agarose, then rotated for 1 hr at 4°C. After washing with the lysis buffer, the antigen was eluted using 200 µg/ml 3×FLAG peptide (Sigma) in TBS, then pre-cleared by centrifuging at 5,000 rpm for 2 min at 4°C. 5×SDS sample buffer containing 10% 2-mercaptoethanol was added to the collected supernatant and defined as a loading sample. These samples were analyzed by western blotting with anti-FLAG M2 antibody and anti-S-tag antibody (Novagen, Madison, WI), or antibodies for HIF-1α and HIF-2α.

### Statistical Analysis

Data were expressed as mean ± standard error (S.E.). Comparisons of parameters among groups were made by one-way analysis of variance (ANOVA), followed by Newman–Keuls’ test. Differences were considered significant at *P*<0.05.

## Results

### HIF-1α and HIF-2α Activate the CD133 Promoter

To reveal the molecular mechanisms of CD133 gene expression, we first used HEK293 cells. A reporter gene assay showed that the P5 basal activity was highest among the five putative CD133 promoters, and overexpression of HIF-1α and HIF-2α increased the activity of all promoters (Fig. 1B). In particular, P5 promoter activity was upregulated approximately 4-fold and 2.5-fold after overexpression of HIF-1α and HIF-2α, respectively. Human colon cancer WiDr cells had an effect similar to that of HEK293 cells (Fig. 1B). Although P5 promoter activity was upregulated by hypoxia, the extent of upregulation was not as enormous as with overexpression of HIF-1α or HIF-2α in HEK293 cells and WiDr cells (Fig. 1C and 1D, respectively).

### The Region between −98 and −25 is Required for Upregulation of CD133 P5 Promoter Activity by HIF-1α and HIF-2α

**Figure 2:**
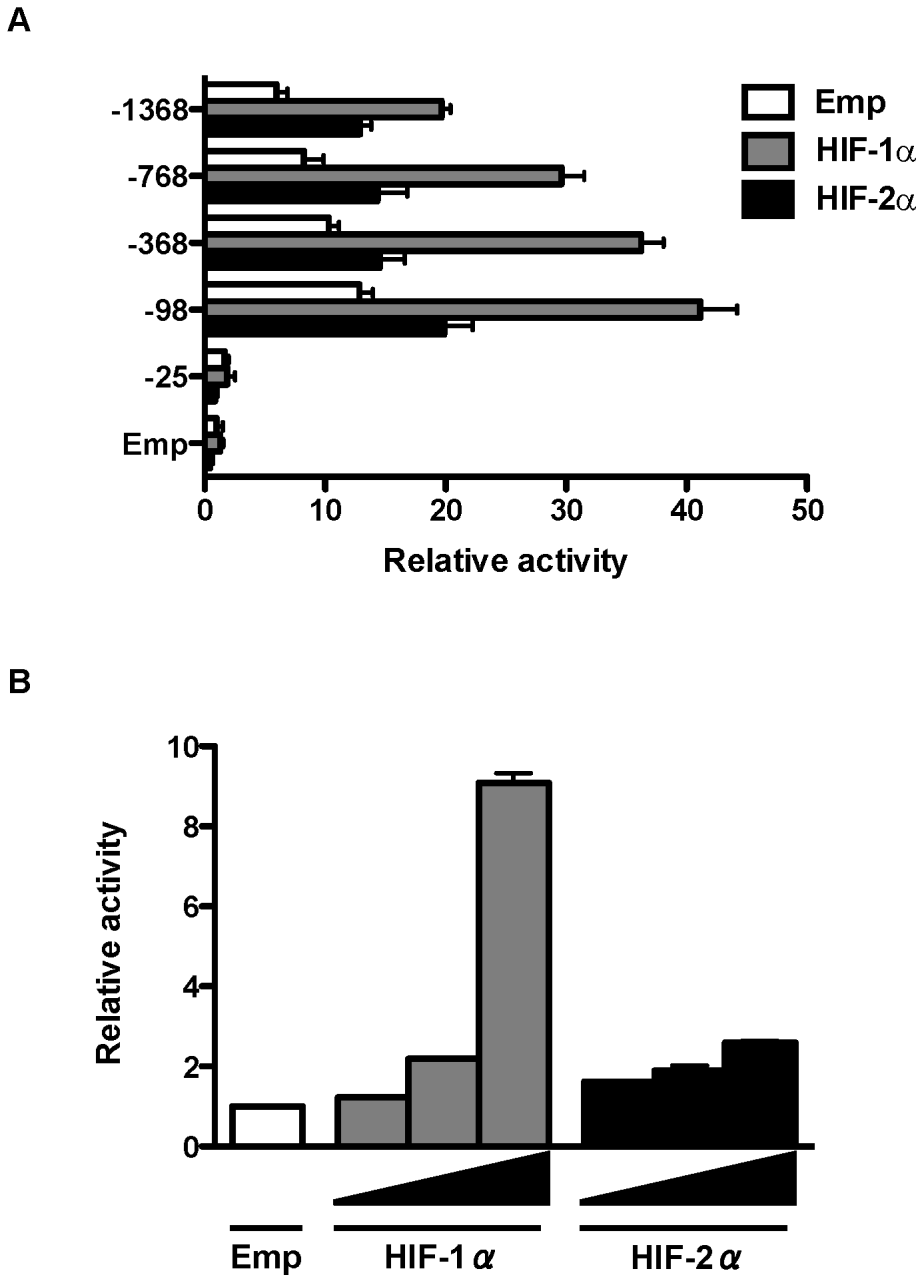
Deletion analysis of CD133 P5 promoter activity and the dose dependent effect of HIFs. (A) Luciferase activities in human embryonic kidney (HEK) 293 cells transfected with a series of deletion mutants of CD133 P5 promoters and HIF-1α or HIF-2α. (B) P5 −98 bp promoter activity of HEK293 cells transfected with a different dose of HIF-1α and HIF-2α.

To determine the region in the P5 promoter essential for HIF-1α- and HIF-2α-induced activation, we performed reporter gene assays using a series of P5 promoter deletion mutants of (pGL3enh-P5−768 bp, −368 bp, −98 bp, −25 bp). Without overexpression of HIF-1α or HIF-2α, deletion to −98 bp demonstrated highest activity; however, further deletion to −25 bp led to a significant reduction in activity (Fig. 2A), suggesting that the region between −98 bp and −25 bp is required for promoter activity. Upregulation of P5 activity by HIF-1α and HIF-2α was maintained until deletion to −98; however, remarkable reduction of the promoter activity was observed by further deletion to −25. HIF-1α and HIF-2α dose dependently increased the P5 −98 bp promoter activity, respectively (Fig. 2B).

### EBS is Required for CD133 P5 Promoter Activity

**Figure 3:**
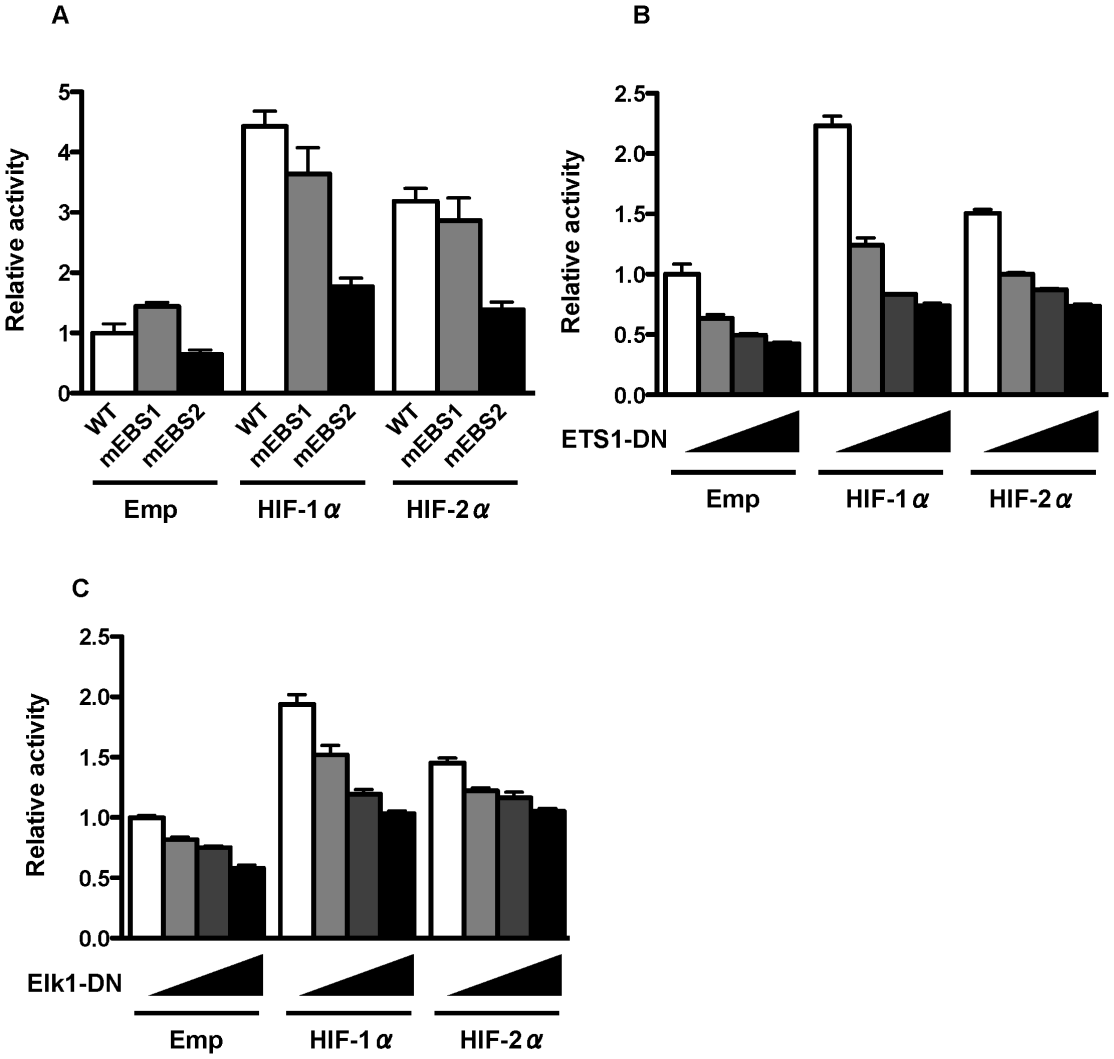
Effect of HIFs and dominant-negative forms of ETS families on the P5 −98 bp promoter. (A) Luciferase activity of the P5 −98 bp promoter with mutation at the two putative EBSs (mEBS1 and mEBS2) after overexpression of HIF-1α or HIF-2α, using human embryonic kidney (HEK) 293 cells. (B, C) Luciferase activity of the P5 −98 bp promoter in HEK293 cells after overexpression of HIF-1α or HIF-2α together with dominant-negative forms of ETS families (ETS1-DN and Elk1-DN).

The region between −98 bp and −25 bp of the P5 promoter contains two EBSs [21], [22]. To determine whether these EBSs are required for promoter activation by HIF-1α and HIF-2α, EBS mutants (mEBS1 and mEBS2) were constructed, and a reporter gene assay was performed. The promoter activity of the proximal site mutant (mEBS2) decreased significantly under overexpression of HIF-1α or HIF-2α (Fig. 3A). To investigate the involvement of ETS-family proteins in this region, two dominant negative ETS mutants (ETS1-DN and Elk1-DN) were constructed, and the promoter activity of P5 −98 bp was analyzed. Promoter activity driven by HIF-1α and HIF-2α was decreased by ETS1-DN and Elk1-DN, respectively, suggesting that HIF-1α and HIF-2α regulate P5 −98 bp promoter activity through ETS-family proteins (Fig. 3B and 3C).

### Expression of ETS-family Proteins is not Affected by Overexpression of HIF-1α and HIF-2α

**Figure 4:**
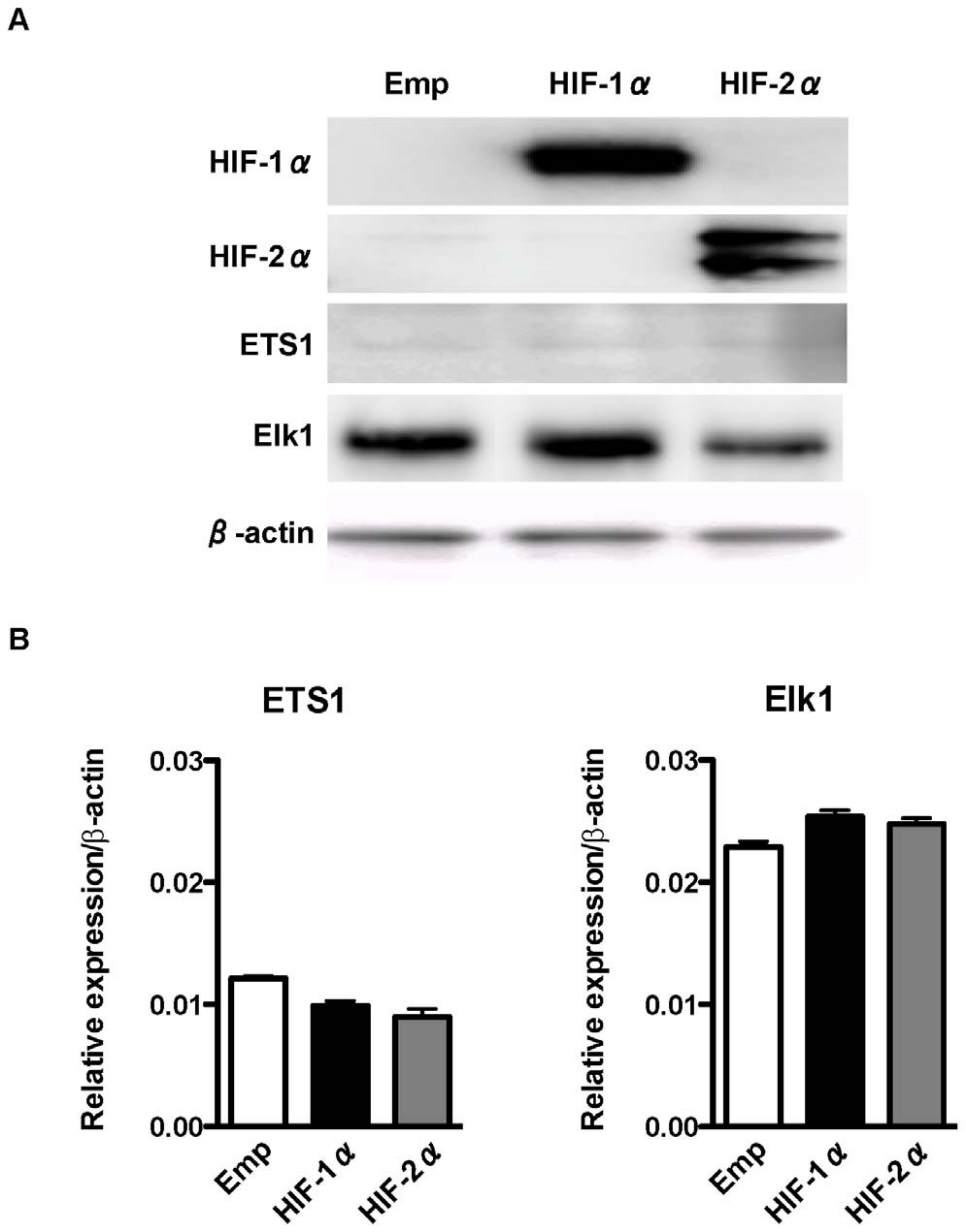
Effect of overexpression of HIFs on the expression of ETS-family transcription factors. (A) Western blot analysis of HIF-1α, HIF-2α, and ETS-family transcription factors after overexpression of HIF-1α or HIF-2α using human embryonic kidney (HEK) 293 cells. β-actin is an internal control. (B) Quantitavive real-time reverse-transcription PCR (qRT-PCR) of ETS-family transcription factors after overexpression of HIF-1α or HIF-2α using HEK293 cells. Data are shown as relative to the expression of β-actin.

Because the P5 −98 bp promoter does not contain an HRE, we hypothesized that HIF-1α and HIF-2α activate the P5 −98 bp promoter through upregulation of ETS-family proteins. However, western blotting (Fig. 4A) and qRT-PCR analysis (Fig. 4B) demonstrated that the expressions levels of ETS1 and Elk1 were not affected after HIF-1α or HIF-2α overexpression, respectively. These results suggest that HIF-1α and HIF-2α activate the P5 −98 bp promoter not by upregulation of ETS-family proteins but through other mechanisms involving ETS-family proteins.

### HIF-1α Binds to the CD133 P5 Proximal Promoter through Elk1

**Figure 5:**
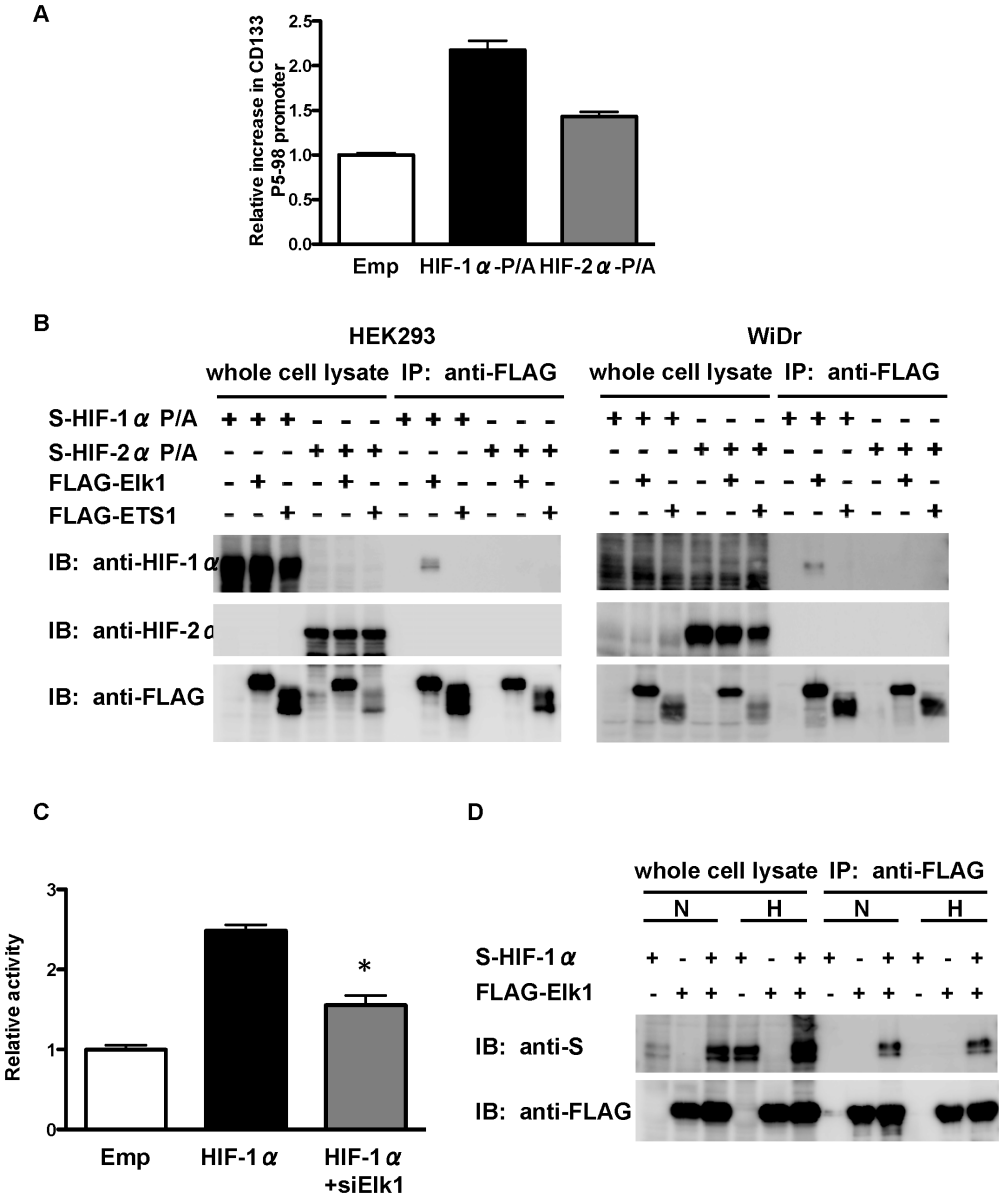
Binding of HIFs to CD133 P5 proximal promoter and ETS-family proteins. (A) Chromatin immunoprecipitation (ChIP) assay showing the binding of O_2_-stable HIF-1α and HIF-2α (HIF-1α-P/A and HIF-2α-P/A, respectively) to the CD133 P5 promoter (between −98 bp and +10 bp) in WiDr cells. (B) IP-western blot analysis showing the binding of HIF-1α-P/A and HIF-2α-P/A to ETS1 or Elk1 using human embryonic kidney (HEK) 293 cells (left) and WiDr cells (right). (C) Luciferase activity of P5 −98 bp promoter in HEK293 cells after overexpression of HIF-1α together with the knockdown of Elk1. *P<0.05 vs. HIF-1α overexpression. (D) IP-western blot analysis showing the binding of HIF-1α to Elk1 under normoxia and hypoxia in HEK293 cells.

Next we examined whether HIF-1α and HIF-2α bind to the CD133 P5 −98 bp promoter through ETS proteins in human colon cancer WiDr cells that express abundant CD133 mRNA and protein. A ChIP assay showed that FLAG-tagged O_2_-stable HIF-1α or HIF-2α mutant bound to the region between −98 bp and +10 bp of the the P5 promoter, which comprises EBS2, more efficiently than to the FLAG-tagged empty vector (Fig. 5A). These results suggest that HIF-1α and HIF-2α bind to the CD133 P5 promoter through ETS proteins. We then used co-immunoprecipitation analysis to investigate whether HIF-1α and HIF-2α bind to ETS proteins in HEK293 as well as WiDr. As shown in Fig. 5B, HIF-1α bind to Elk1, but not ETS1 in both HEK293 and WiDr. However, HIF-2α did not bind to Elk1 or ETS1. Upregulation of the P5 promoter by HIF-1α was significantly suppressed by knockdown of Elk1 (Fig. 5C). Hypoxia did not influence the amount of HIF-1α and Elk1 binding (Fig. 5D).

### Proximal P5 Promoter Activity and Expression of CD133 were Regulated by HIF-1α and HIF-2α in the CD133-positive Colon Cancer Cell Line WiDr

**Figure 6:**
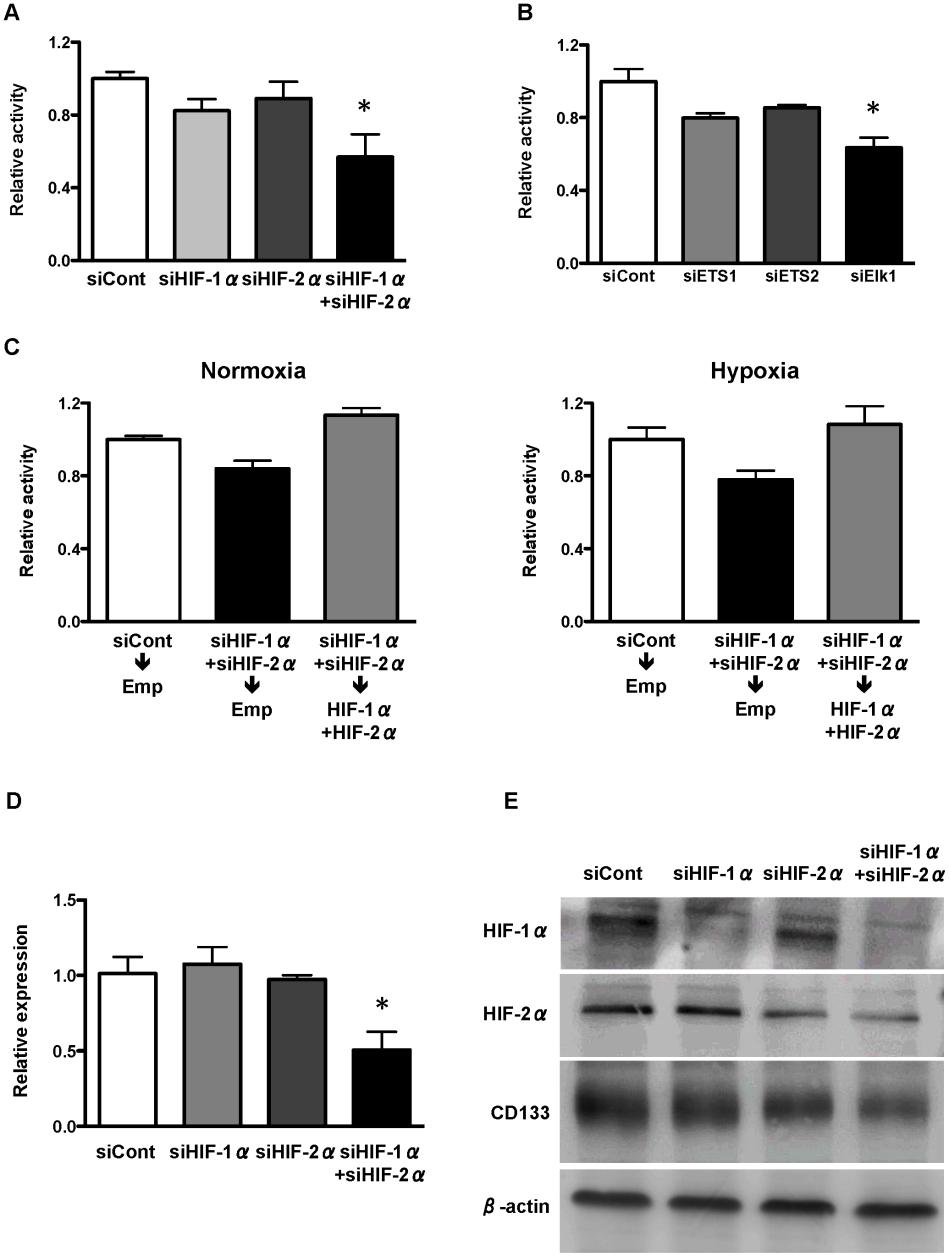
Effect of HIFs or ETS knockdown on CD133 promoter activity and expression in WiDr cells. (A) P5 −98 bp promoter activity under knockdown of HIF-1α and HIF-2α. (B) P5 −98 bp promoter activity under knockdown of ETS families. (C) P5 −98 bp promoter activity after overexpression of HIF-1α and HIF-2α 24 hrs after knockdown of HIF-1α and HIF-2α under normoxia and hypoxia. (D) Quantitative real-time reverse-transcription PCR (qRT-PCR) analysis of CD133 under knockdown of HIF-1α and HIF-2α. *P<0.05 vs. control siRNA (siCont). (E) Western blot analysis of HIF-1α and HIF-2α and CD133 under knockdown of HIF-1α and HIF-2α. β-actin is an internal control.

To verify the mechanisms observed in HEK293 cells, we repeated our expreriments using WiDr cells. A reporter gene assay was performed on the P5 −98 bp promoter, which showed that promoter activity was significantly decreased by knockdown of both HIF-1α and HIF-2α (Fig. 6A). Furthermore, knockdown of Elk1, but not ETS1, significantly decreased the P5 −98 bp promoter activity (Fig. 6B). Decreased activity of P5 −98 bp promoter by the knockdown of both HIF-1α and HIF-2α was recovered after overexpression of both HIF-1α and HIF-2α (Fig. 6C).

To confirm the effect of HIF-1α and HIF-2α on CD133 transcripts and proteins, qRT-PCR and western blotting were conducted under normoxic conditions. Consistent with the results of the reporter gene assay, expression of CD133 mRNA and protein decreased when both HIF-1α and HIF-2α were knocked down (Fig. 6D and 6E, respectively).

## Discussion

In this study, we focused on the regulation of CD133 by HIF-1α and HIF-2α, and demonstrated that 1) HIF-1α and HIF-2α upregulate CD133 promoter activity, particularly of P5, 2) HIF-1α and HIF-2α bind to the proximal CD133 P5 promoter at EBS, 3) HIF-1α physically interacts with Elk1, and 4) expression of CD133 is regulated by HIF-1α and HIF-2α in colon cancer cells.

Among the five alternative promoters of CD133, P1 has been reported to be most strongly associated with hypoxia-induced promoter activity and gene expression of CD133 in lung cancer cell lines [20]. In addition, Oct3/4 and Sox2, both of which are induced by HIF-1α and HIF-2α, promoted CD133 expression through their direct interaction with the P1 promoter. In contrast, our observation demonstrated that P5, but not P1, had the highest upregulation by the overexpression of HIF-1α and HIF-2α in HEK293 cells and WiDr (Fig. 1C and D). We speculate that these differences are due to the use of different cell lines, different hypoxic conditions (0.1% vs. 1%), and different P1 promoter constructs; the length of P1 promoter was rather longer than that used in this study (1800 bp vs. 1368 bp) [20]. In line with our results, it has also been reported that P5 activity was highest in the colon cancer cell line Caco-2 [21]. Therefore, we focused on the regulation of P5 promoter activity by HIF-1α and HIF-2α independent of hypoxia.

Our results suggest that HIF-1α and HIF-2α are involved in transcriptional regulation of CD133. However, P5 does not comprise an HRE, but comprises two EBSs instead.

Our results are consistent with those of a previous study in which overexpression of ETS2-DN and Elk1-DN significantly decreased the P5 promoter activity in colon cancer cells [21]. The ETS family includes nuclear phosphoproteins involved in many biological processes, such as cell growth, differentiation and survival [23]. Recently, it has been reported that HIF-1α and HIF-2α physically and functionally associate with ETS-family transcription factors. For example, ETS variant-4 (ETV4), a member of the ETS family of proteins, can activate the prolyl-4-hydroxylase domain 2 (PHD2) promoter in cooperation with HIF-1α through the HIF binding site [24], and HIF-2α activates the VE-cadherin promoter independently of hypoxia and in synergy with ETS1 through EBS [25]. In addition, glutathione S-transferase (GST) pull-down assay demonstrated that HIF-2α physically interacts with ETS1 [26], and immunoprecipitation analysis showed that HIF-2α forms a complex with Elk1 in MCF7 (breast cancer) and 786-O (renal cell carcinoma) cells [27]. Therefore, we hypothesized that HIF-1α and HIF-2α regulate CD133 promoter activity through ETS-family transcription factors. In the present study, EBS2 located in the region between −98 bp and −25 bp was found to be essential for HIF-induced activation of the P5 promoter. Additionally, a ChIP assay showed that HIF-1α and HIF-2α bind to the proximal P5 promoter harboring EBS2 and HIF-1α physically interacts with Elk1. These data strongly suggest that HIF-1α transcriptionally activates the P5 promoter through EBS2 by forming a complex with Elk1. Although we could not detect a physical interaction between HIF-2α and ETS1 or Elk1 (Fig. 5B), a ChIP assay showed that HIF-2α binds to the proximal P5 promoter harboring EBS (Fig. 5A), suggesting that HIF-2α regulates the P5 promoter through interaction with other ETS-family proteins. Further analysis is required to identify the ETS-family transcription factors that are involved in HIF-2α-meditaed activation of the P5 promoter.

Our observations suggest that HIF-1α and HIF-2α regulate CD133 transcription; however, promoter activity was not significantly upregulated by hypoxia. Unlike our findings, it has been reported that hypoxia downregulated CD133 transcription and that mTOR signaling and HIF-1α are involved in regulating CD133 expression [28]. Although the reason for this is unclear, it is possible that HIFs are regulated by other mechanisms, such as ras-mitogen-activated protein kinase (RAS-MAPK) signaling. Recently, the ras/extracellular singnal-activated kinase (RAS/ERK) signaling pathway has been demonstrated to promote CD133 transcription in colon cancer cells [21]. In addition, inhibition of the phosphatidylinositol 3-kinase (PI3K)-Akt or ERK1/2 pathway reduced the hypoxia-driven CD133 expansion in glioma cells [19]. HIF-1α can be stabilized not only through hypoxia, but also through oncogenic signaling pathways including RAS, and ERK1/2 can directly phosphorylate HIF-1α [29], [30]. HIF-1α has also been shown to possess a MAPK docking domain and to bind to ERK2 [31]. Therefore, our observation could imply that the RAS/ERK signaling pathway promotes CD133 transcription through HIF-1α and HIF-2α, independently of hypoxia.

Recently, it has been demonstrated that the expression of CD133 was upregulated under hypoxia in a HIF-1α-dependent manner in pancreatic cancer cells, and knockdown of HIF-1α partially abrogated the elevated CD133 expression under hypoxia [32]. In addition, hypoxia promoted expansion of the CD133-positive glioma stem cells through activation of HIF-1α, and the CD133 expression level was increased under the chemical hypoxia in renal cancer cell lines [19], [33]. Furthermore, hypoxia induced CD133 expression in human lung cancer cells by upregulation of Oct3/4 and Sox2 through HIF-1α and HIF-2α [20]. In the present study, however, hypoxia did not influence the expression of CD133 in WiDr cells (data not shown), and upregulation of P5 promoter activity under hypoxia was not as significant as with overexpression of HIF-1α or HIF-2α in HEK293 and WiDr cells (Fig. 1B-D). Furthermore, knockdown of both HIF-1α and HIF-2α under normoxia downregulated the expression of CD133 in WiDr (Fig. 6D and 6E). These results suggest that HIF1-α and HIF-2α regulate the promoter activity and expression of CD133 independently of hypoxia in colon cancer cells. In accordance to our results, it has also been demonstrated that HIF-1α enhances tumor-initiating cell frequency *in vivo* in part by regulation of the expression of CD133 and the Notch pathway in breast cancer cells [34].

Taken together, the results of the present study suggest that HIF-1α and HIF-2α regulate the expression of CD133 by controlling CD133 promoter activity, possibly through ETS proteins. Elucidating the mechanisms underlying transcriptional regulation of cancer stem cell markers such as CD133 may lead to the development of a novel target to eradicate CSC.
